# A Review of Fusible Interlinings Usage in Garment Manufacture

**DOI:** 10.3390/polym10111230

**Published:** 2018-11-06

**Authors:** Qian Zhang, Chi-Wai Kan

**Affiliations:** Institute of Textiles and Clothing, The Hong Kong Polytechnic University, Hung Hom, Kowloon, Hong Kong, China; hina.zhang@connect.polyu.hk

**Keywords:** fusible interlining, garment manufacture, printable interlining

## Abstract

The consumer’s enhanced awareness of garment quality, appearance, and related eco-safety production manufacture means that not only shell fabrics, but also accessory materials should be paid much more attention. Interlining is the one of the most important and state of the art accessory materials that currently lacks review and exploration. This article comprehensively demonstrates an organizational integration of interlinings which includes their history, classification, manufacture, characteristic, properties, function, fusing technology and application. In addition, the article highlights a new innovation of printable interlining, which could replace the traditional fusible interlinings because of its cost-effectiveness, its simple process and its environmentally-friendly nature.

## 1. Introduction

Interlining is a layer of fabric inserted between the shell fabric and the lining of a garment to give clothing a suitable appearance and stability. It has a long history and exists in diverse forms based on its substrate and application. The most well-known and widely used interlinings are fusible interlinings and this article mainly focused on fusible interlinings.

Although previous research articles concerning the properties and functions of shell fabrics fuse different interlinings by suitable fusing technology [1,2,3,4,5,6,7], there is a lack of integral comprehension of the knowledge of fusible interlinings. This review article will systematically summarize state-of-the-art progress in the history, classification, manufacture, characteristics, properties, function, fusing technology and application of these materials. Among the abundant research work devoted to interlinings, the big challenge of extending their properties into better quality and cost-effectively fabric has long been recognized as the core issue for practical applications. Researchers never stop their forward process on the way to the new areas of material and technology, and this has led to printable interlinings being created in place of traditional fusible interlinings. The major knowledge and techniques are reviewed here in order to give helpful guidance for future research [8,9,10].

## 2. History

Interlining is a kind of accessory material that is a crucial element influencing the quality and performance of garments and furthermore has effects on consumer purchases. Interlining has a long history, it has experienced a long process of classification extension, production technology improvement, and function development. Table 1 shows the details of interlinings’ evolution.

## 3. Interlining Classification

Based on the application method, there are two kinds of interlinings: Sewn interlining and fusible interlining. The interlining which could be fixed with the garments components by sewing is called sewn interlining. For the preparation of sewn interlining a piece of fabric is treated with starch and allowed to dry and finally sewn with the main fabric. In addition, the fusible interlining which could be fixed with the garments components by applying heat and pressure for a certain time is called fusible interlining. Compared with sewn interlinings, the overall performance of fusible interlinings is better, and the fusing technique is easier. The interlining in this article refers to fusible interlining [26].

Basically, the classification of interlinings is based on the base cloth (substrate). A substrate is an interlining material onto which the thermoplastic resin is coated, sprayed or printed. There are different types of interlinings: (i) woven interlining; (ii) non-woven interlining; (iii) knitted interlining; (iv) non-general interlinings [25,26,27]. 

### 3.1. Woven Interlining

**Figure 1:**
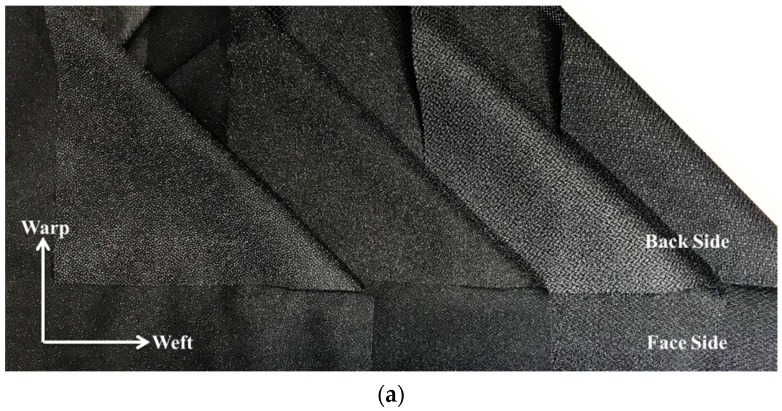

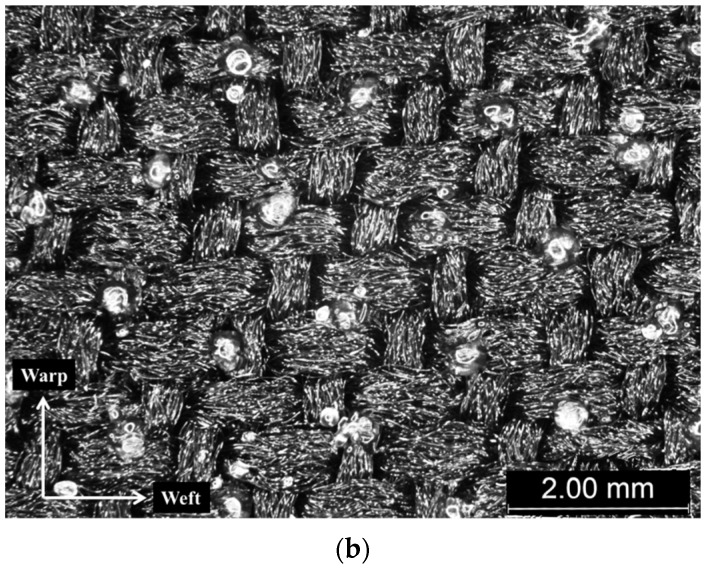
Woven Interlining (**a**) and Microscope Views of Woven Interlining (**b**).

Woven interlining is a woven substrate base of fusible interlining (Figure 1). Woven interlinings are used for different zones along the garment warp. Each zone possesses different properties in terms of handle, drape, and resilience. Woven interlinings made from lightweight fabrics usually used for the most demanding conditions like jackets, waistband, outerwear plackets, collars and front shirt bands etc. An outstanding example of this application is an interlining with a soft handle in the warp and a firm handles in the weft. This interlining is specifically produced for the fronts of men’s jackets and provides a firm basis for the length of the front and also prevents the lateral sections across the chest and shoulder from collapsing [28,29,30,31]. Woven interlinings support a very good performance of the garment. However, it has a relatively high cost in comparison with other types available.

### 3.2. Non-Woven Interlining

**Figure 2:**
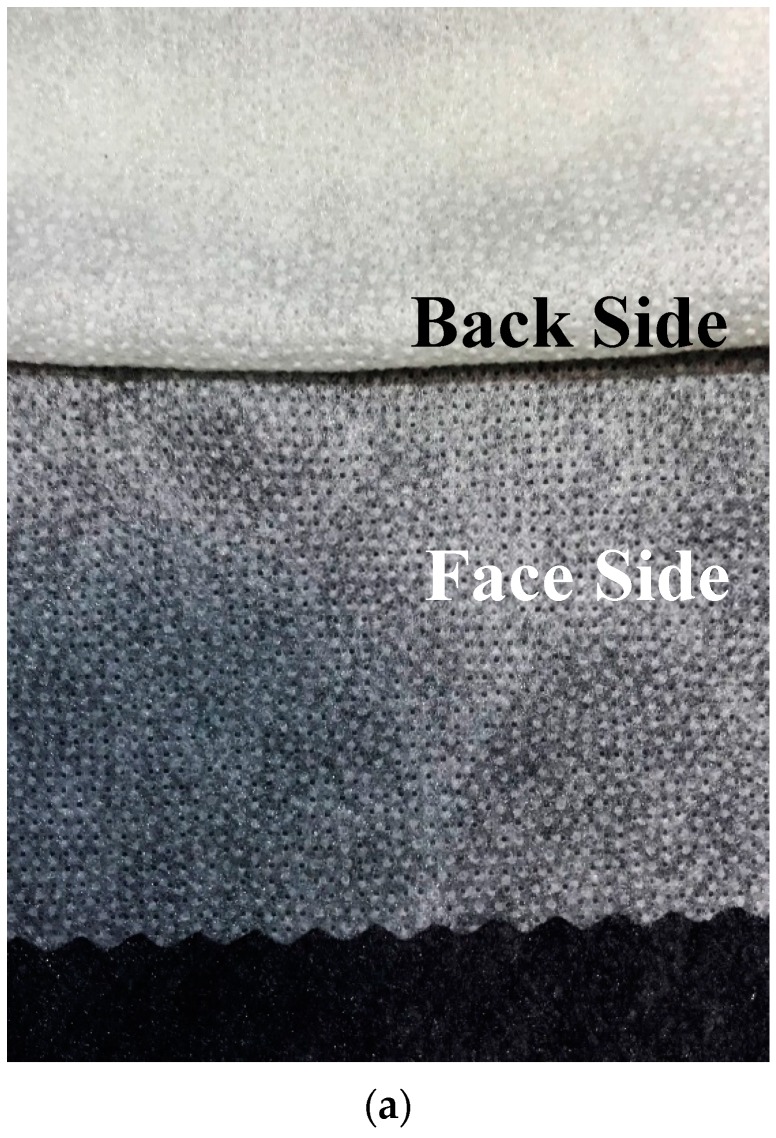

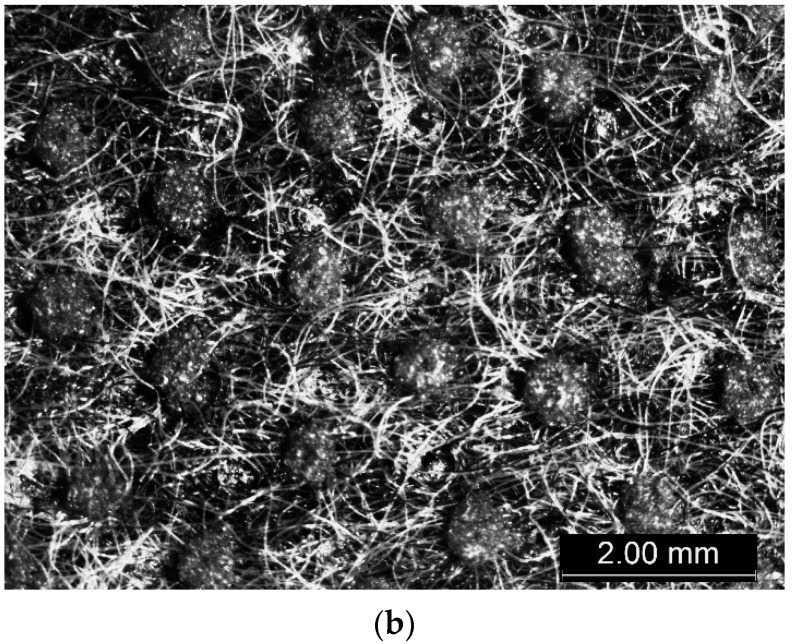
(**a**) Non-woven interlining; (**b**) microscope views of non-woven Interlining.

Non-woven, as the name suggests is not formed through the textile cloth (Figure 2). A non-woven substrate is a series or mixture of fibers and construction includes random, parallel, cross and composite types. Non-woven interlinings are generally less expensive than woven or knitted fabrics due to the method of fabrication. High quality non-woven interlinings are made from 100% polyamide products with ultra fine coating to heavier blends. These are thermally or chemically bonded and used depending on applications. The non-woven interlinings used by clothing manufacturers originate from the paper industry and their development was brought about by shortages of traditional fabrics [27,32,33].

### 3.3. Knitted Interlining

**Figure 3:**
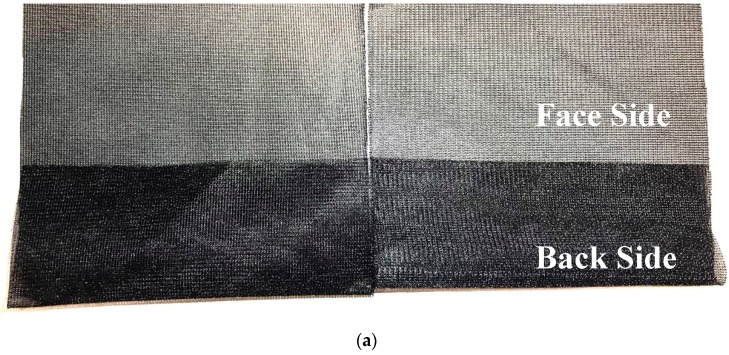

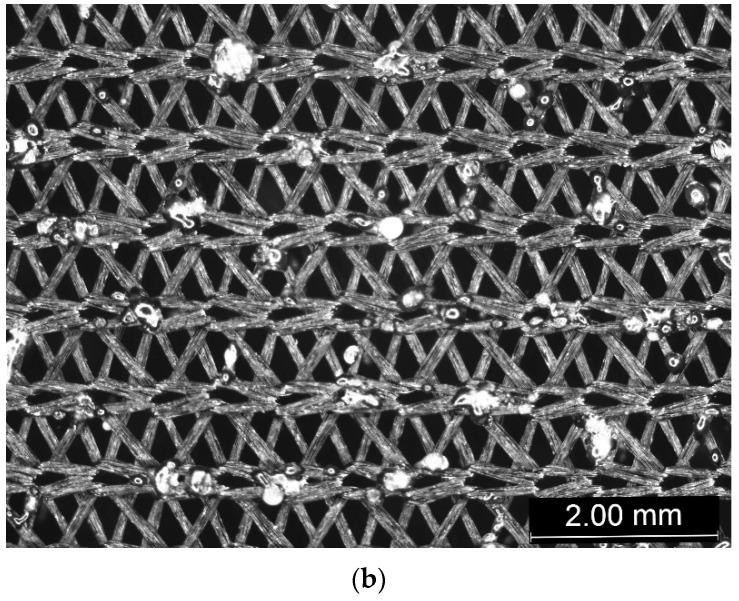
(**a**) Knitted interlining; (**b**) microscope views of knitted interlining.

Fusible knitted interlinings are basically used in knit garments providing the perfect stretch basis for efficient production (Figure 3). Circular and jersey knit fusible interlinings have stretch and recovery properties which used in stretchable merged areas. Because of knitted interlining construction, it provided a considerable degree of elasticity to the fused components by yielding easily to body and limb movements. The growing use of knitted fabrics for men’s and women’s wearing led to the introduction of knitted interlinings in the early 1960s [34]. The first products were warp-knitted and it was used until the introduction of weft-inserted fibers into the warp-knitted construction that this type of interlining became widely accepted as a fusible for woven cloths. The fabrics used as base fabrics for fusible interlinings are warp knitted and the two types of construction in general use are lock nit and weft insert [35,36]. The advantage of a weft-inserted interlining is that it has a natural handle while providing resilience in the weft direction, i.e., around the body. The newest weft-inserted types include a stretch property in the weft fiber that provides extensibility in this direction and thus makes it more compatible with the outer fabric. The warp-knitted interlinings are widely used for woman’s light clothing, such as blouses and dresses made of crepe-de-chine, georgette, and polyester-fiber yarns [5,37].

### 3.4. Non-General Interlinings

#### 3.4.1. Water Repellent Interlinings

**Figure 4:**
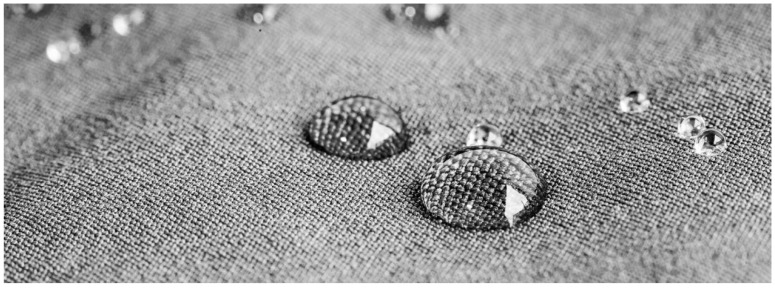
Water repellent interlinings.

Water repellent interlinings (Figure 4) are thermally bonded, the base fabric are usually non-woven and circular knits which can withstand the rigors of commercial wash processes. These are specifically designed for rainwear piece productions [38].

#### 3.4.2. Hair Interlinings

**Figure 5:**
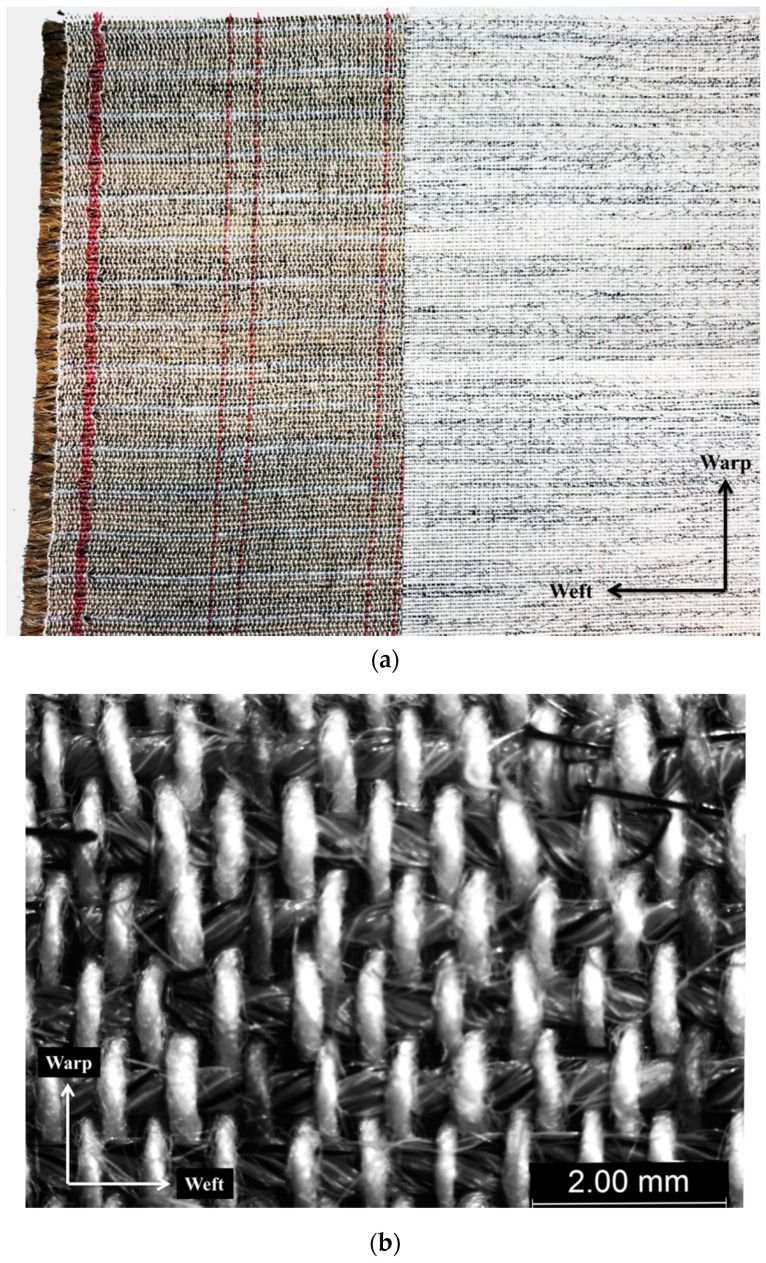
Hair interlinings (**a**) and Microscope Views of Hair Interlining (**b**).

Hair interlinings are woven canvas made from horse hair mostly used in men’s formal jackets and blazers etc. (Figure 5). Basically, there are two types of applications: Fusible and non-fusible (sew-on). The former is attached to the backside of the shell fabric through thermal bonding or fusing while the latter is attached by stitching. Using strengthened interlinings can provide stability to the shell fabric, reduce creases, improve drape and firmness to the shell fabric [39].

## 4. Manufacture of Interlining

Fusible interlinings are fabrications coated with some form of resin or adhesive that serves as a bonding resin to hold the interlining to the shell fabric. Substrates may be woven, knits, or fiber webs [40,41].

**Figure 6:**
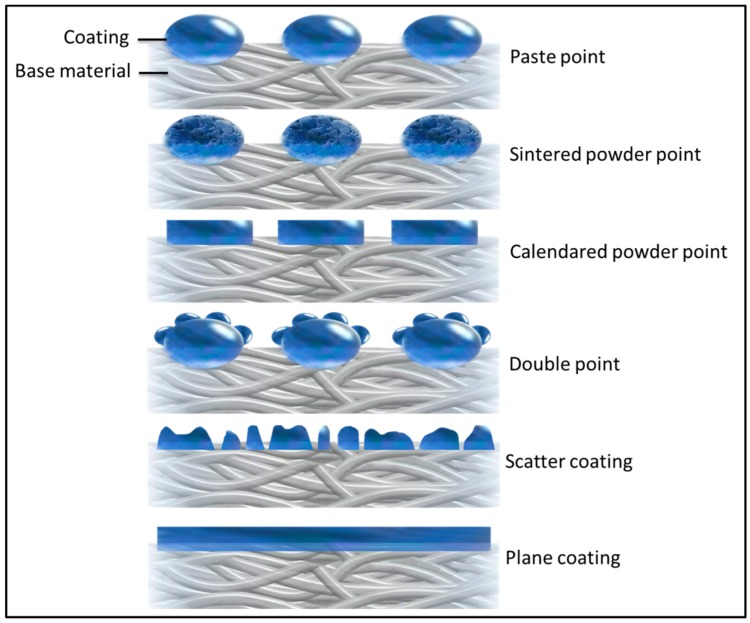
Different coatings.

**Figure 7:**
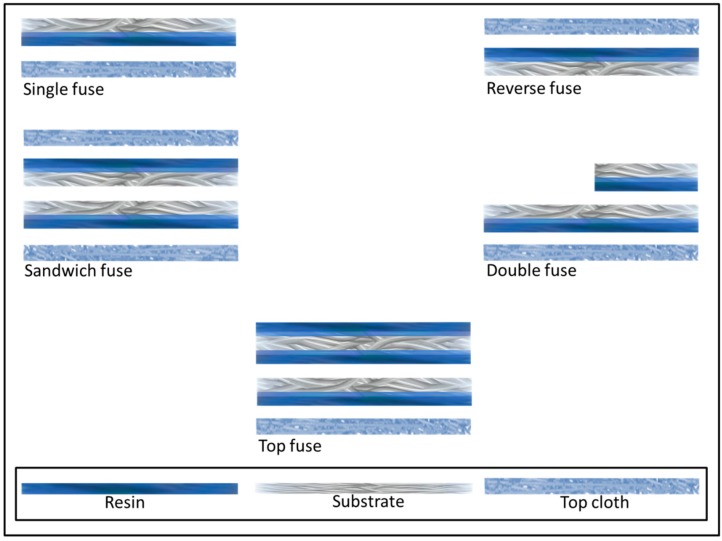
Fusing methods.

In the coating process, an adhesive chemical is applied to the fabric in a wide variety of forms. In the apparel application, the high-quality products are coated in a dot formation. One has concentrated on this type of coating to avoid much of the surface area of the product from being saturated with the adhesive chemicals. The dot formation keeps the surface coverage with adhesive to its ultimate necessary minimum. The adhesive substance consists of either powder or paste. The powder dot is applied with heat, thereby melting the adhesive while it is applied. The paste dot is applied in viscous form before the water content is evaporated in a long heat chamber to leave a solid dot. There are many different types of coatings, including paste point, sintered power point, calendared powder point, double point, scatter coating and plane coating which shown in Figure 6. In addition, the fusing method is a variable, which has shown in Figure 7.

## 5. Characteristics of Interlining

There are three basic characteristics of interlinings that are (i) fiber content, (ii) weight, (iii) fabrication and (iv) adhesive points. These factors contribute to the aesthetics and performance of interlinings.

### 5.1. Fiber Content

Fiber content contributes to the strength, hand, weight, and resiliency of interlining [42]. Any fiber, or combination of fibers, can give a distinct handle type and by changing one or more of these variables, an entirely new product can be developed. Changes to staple length, denier or fiber finish can be made to the end product and influence the final interlining. Depending upon the end application, properties such as drape, softness, firmness, fullness, recoverability and durability can be designed into the end product. In order to make a choice of fiber, the end application must be borne in mind [43,44]. The fibers used for woven, knitted and non-woven substrates are owing to the cost of fibers, whether natural, synthetic, or a mixture of both. The great majority of common fiber types are man-made fibers including polyester fiber, polyamide fiber, acrylic fiber, viscose rayon, nylon, or any combination of these [42,43,44,45,46,47,48,49].

### 5.2. Weight

Interlinings are available in a wide range of weight from 13.56 to 135.62 gram per square meter [50]. Heavier interlinings provide more support for heavier, more structured apparels such as coats and suits. Lighter-weight interlinings offer resiliency and some support, and they may provide a softer hand [51]. In recent years, fashion trend has been to softer materials and less structured look in men’s and women’s clothing, however, it may decrease the stability and resiliency of garments.

### 5.3. Fabrication

Interlinings are available in four basic fabrications: (i) fiber web; (ii) woven; (iii) knit; and (iv) foam lamination [52].

#### 5.3.1. Fiber Web

Fiber webs are a widely used fabrication for interlining due to low cost, versatility, and the ease of engineering specific characteristics into the interlining. Fiber webs may have less strength but do not ravel in handling, which is a benefit during sewing operations. The performance of interlinings made of fiber webs is very closely linked to the fiber content, fabric weight, and the fiber orientation in the web. Fiber web interlinings are most often found in washable garments [9,19]. Pilling is a problem often associated with fiber webs since fiber webs tend to have low abrasion resistance [53]. The basic methods of orientating the fibers that form the substrate or web include dry-laid web formation, wet-laid web formation, and the air-laid method [54].

#### 5.3.2. Woven

Normally fusible base clothes are woven in plain weave, that is, where warp and weft interlace under and over alternate threads giving the most compact and stable construction. Woven interlinings may be produced from almost any type of fiber. This fabrication is usually the most expensive and subject to raveling and shrinkage. Woven substrates can be produced from animal hair, viscose rayon, cotton, polyester fiber, acrylic fiber, wool, or in any number of combinations of these fibers [29].

#### 5.3.3. Knit

Knit fabrics used for interlining are primarily warp knit tricots, raschels, and weft insertion raschels. The tricots and raschels used for interlining purposes are produced in varied weights with high stability and little or no stretch. They are used for strength in relation to weight, low bulk, and smooth hand [55]. Knits structure contribute to strength, and resiliency without adding bulk and weight. Since the knitting process is generally faster than weaving, the cost of knitted substrates is somewhat lower than that of an equivalent woven product [56].

#### 5.3.4. Foam Lamination

Foam substrates may be laminated to shell fabrics or linings to improve body and increase stiffness, durability and warmth. The conception of laminating flexible substrates is based on either water or solvent adhesives [57]. Here the adhesive is applied as a thermos-fusible resin in the form of a dot coating that imparts greater flexibility to the laminate, factors important in subsequent molding applications [58]. Foam, which also provides insulation, may be used as an interlining on moderate-priced cloth coats [56].

### 5.4. Adhesive Points

An interlining has been commonly used to give garments a suitable appearance and stability. The interlining is considered to be the most important subsidiary material. An adhesive interlining which uses thermoplastic resin is a representative [59]. Because the properties of a shell fabric were considerably changed by laminating adhesive interlining, and this has affected the garments’ properties, studies about the effect of adhesive interlining on laminated fabric are necessary [60]. Interlinings are available with indifferent adhesive points. By using the thermoplastic resin to bond, adhesive interlinings are the representative material to give clothing a suitable appearance and stability [21]. Therefore, quantifying the effect of an adhesive point is desirable. Some researchers have investigated the efficiency of adhesive points. In particular, the prediction of mechanical properties for laminated fabric bonded with adhesive interlining is of great interest [61,62,63]. The adhesive method of fusible interlining affects the fabric’s physical properties. Bubbles and strike through and back would have occurred when there was excessive adhesive or unreasonable adhesive conditions [3,64].

## 6. Properties of Interlining

### 6.1. Physical Properties

Fusible interlining generally gives a high level of quality in a garment. This may due to the physical and mechanical property changes of fabric. Physical properties of fusible interlining include style properties, utility properties, durability properties and product production which are summarized below:

#### 6.1.1. Style Properties

Style properties are those changes which affect the emotional appeal. Shell fabric with fusible interlinings has a significant change on a three-dimensional shape, drape of a garment and fabric handle. The application of fusible interlinings would affect the drape of the face fabric, with several interlining types exhibiting a different drape configuration that could be attributed to the physical structure of the fusible interlinings [29]. The effect of sewing and fusing of interlining on drape behavior of men’s suiting fabrics is investigated. Drape coefficient changes with the types of seams and stitches used, as well as with the interlining used [65].

#### 6.1.2. Utility Properties

Utility properties are changes in the wearing function of a garment of shell fabrics with fusible interlinings. Fusible interlinings mainly affect the fabric elongation and air-permeability properties. Application of fusible interlinings affected the shell fabrics’ ability to recover from creasing [29]. Akcagun et al. investigated fusible interlinings decreased air-permeability of wool fabrics [66]. Urbelis et al. found the stress relaxation is changing depending on components’ structure of fusible interlinings [37] It was found that after the deformation of 15% was reached (while testing the relaxation was stretched by 10, 15, and 20%) the more significant stress part is being taken by fusible interlining, and its behavior mostly influences the system’s instantaneous elasticity as well as max stress value during the relaxation testing [37]. Kim et al. investigated and controlled the fabric rigidity, fusible interlining with a thermoplastic agent, is usually used subsequently to give cloth to suitable rigidity and to keep the garment shape [63].

#### 6.1.3. Durability Properties

Durability properties are the capacities of fabric to maintain the style and utility properties during wear. Shell fabrics with fusible interlinings are mainly focused on launder ability and dry- cleaning durability. Koening et al. found that fusible interlinings exhibited some shrinkage in both directions and separated from the face fabric after five laundering cycles [67,68]. Kamat et al. found knitted fusible interlinings shrank significantly in the wale direction during laundering, and the appearance was affected significantly by laundering [19]. Wang et al. studied and tested the shrinkage rates of different fabrics under different fusible interlining conditions as well as those for the same fabric fused to different interlinings. Despite it generally recognized that the thicker the fabric, the smaller the heat shrinkable rate, the statement was found to not be accurate [69].

#### 6.1.4. Apparel Production

Apparel production working performance affects the quality and cost of garments. Fusible interlinings produced a distinct manner of surface distortion of the shell fabric [29]. It also affects the sewing technology and performance. Pasayev et al. investigated the bonding of interlining onto the sewing area helps reduce the seam slippage and the seam slippage was determined to change depending on the interlining type used [70]. Matsunashi et al. studied the behavior of needle penetration in blind stitch sewing and examined cases where interlining is seamed together with other fabrics [71,72].

### 6.2. Mechanical Properties

Mechanical properties of shell fabric with different fusible interlinings were investigated and analyzed by researchers. Kawabata Evaluation System of Fabric (KESF) is a popular fabric objective measurement system. Here low-stress mechanical properties include tensile, bending, shearing, surface and compression. Total 16 low-stress mechanical properties can be tested which is shown in Table 2 [73]. Zhang et al. compared the low-stress mechanical properties of woollen fabric with and without interlining which shown in following sections [7].

#### 6.2.1. Tensile

**Figure 8:**
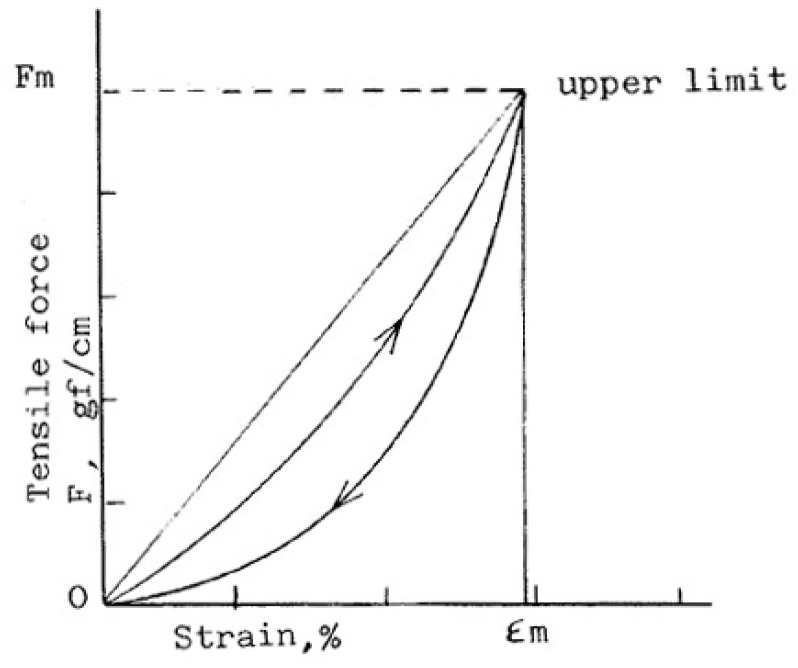
A typical force-extension curve of woven fabric.

**Figure 9:**
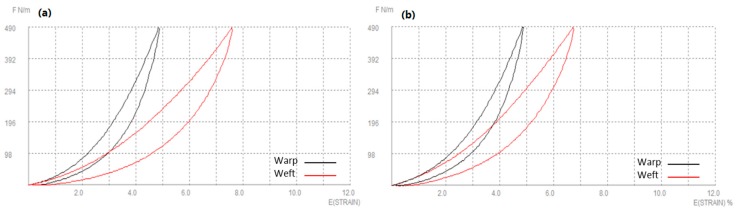
Tensile property of (**a**) woollen fabric; (**b**) woollen fabric with interlining.

Tensile properties depend on the linear density of the thread, the number of warp and weft yarns and the crimp. Figure 8 shows force-extension curve of woven fabric [73]. They are important when determining the drape, hand, sewing, wrinkling and stability of fabrics [4]. Nagano discovered that one of the most important properties changes of attending fusible interlining is a tensile extension [74]. Tensile properties of fused systems were investigated in longitudinal and transverse directions (in respect to outer fabrics warp direction) and on the basis of obtained results recommendations were given to achieve the desired mechanical reaction of fused textile system [28,75]. After fusing outer fabric and fusible interlining into a textile system, linearity increases due to an adhesive layer, which makes system stiffer and decreases tensile strain [35,60]. Figure 9 shows the tensile property of a woollen fabric without interlinings (a) and with interlining (b). From the fabric extension treatment, it is obviously reflected, woolen fabric become stiffer and reduces the elasticity after fusing interlining [7].

#### 6.2.2. Bending

**Figure 10:**
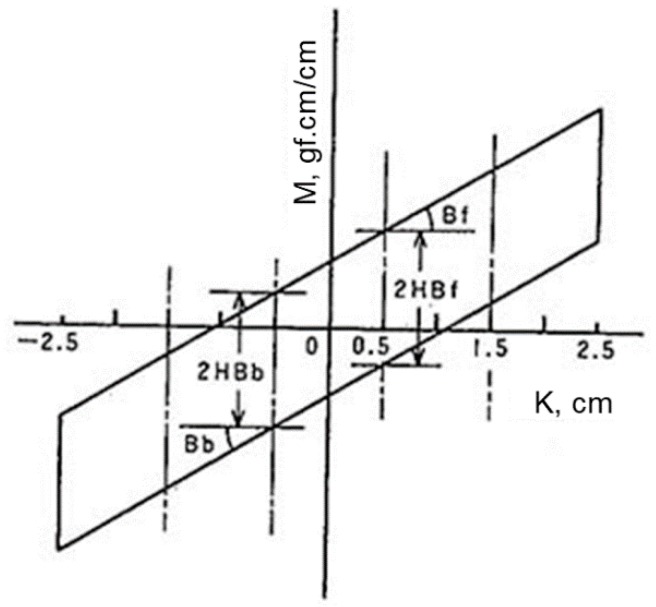
Bending property

**Figure 11:**
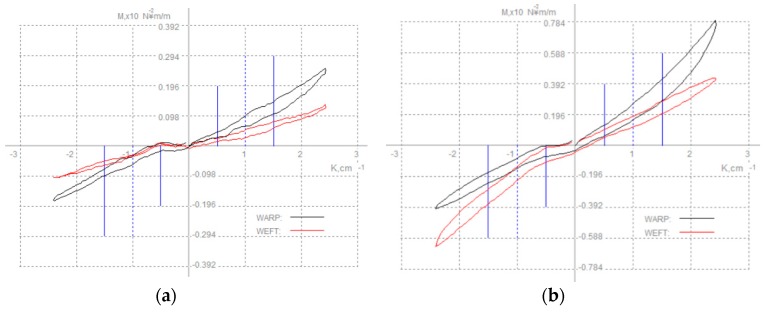
Bending property of (**a**) woollen fabric; (**b**) woollen fabric with interlining.

Bending properties of shell fabrics with fusible interlinings include bending rigidity and bending hysteresis. This property indicated the flexibility and bending recovery ability of the fabric (Figure 10) [73,76]. Some researchers showed that the presence of fused interlinings produced increased bending rigidity of the composite fabric for shape retention [65,77,78,79]. Zhang et al. found interlining adhesive density affects the ability of resistance to bending. In addition, interlining thickness and weight affect bending recovery ability [7]. Figure 11 shows bending property of woollen fabric (a) and fused with interlining (b). It reflects that both B and 2HB of bending property of woollen fabric have significant affected by interlining. Interlining increases their stiffness greatly and decreases the recover properties significantly [7]. 

#### 6.2.3. Shearing

**Figure 12:**
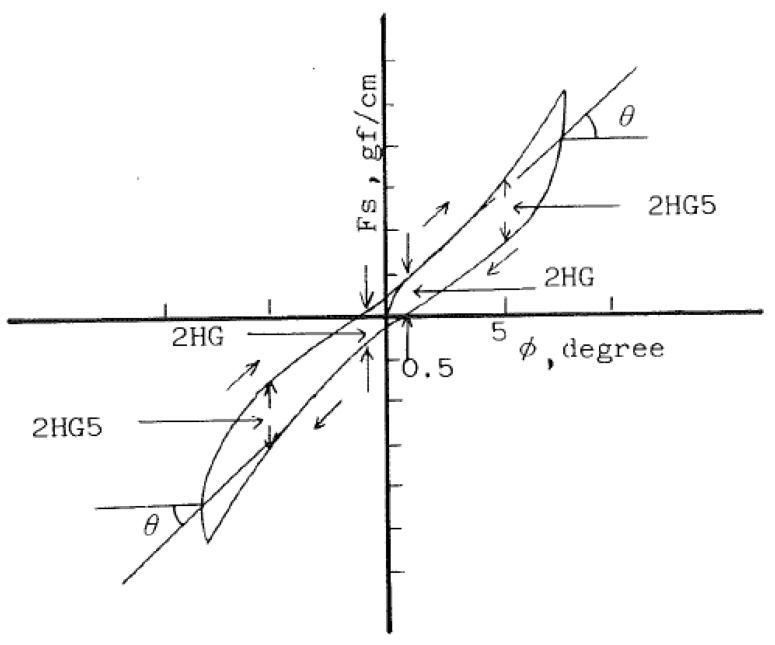
Typical shear force-shear angle curve of woven fabric.

**Figure 13:**
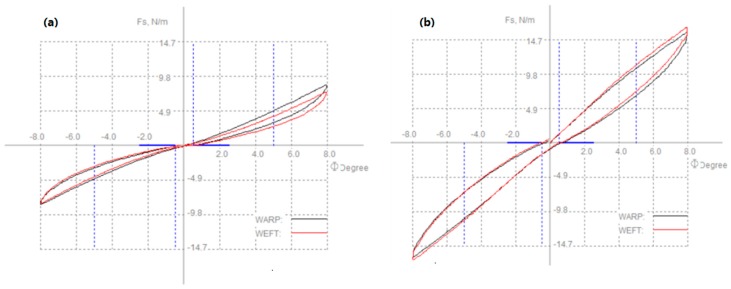
Shearing property of (**a**) woollen fabric; (**b**) woollen fabric with interlining.

Shearing properties measured shear stiffness, shearing hysteresis which defined the ability of a fabric to resist shear stress [80]. Figure 12 shows a shear force-shear angle curve of woven fabric [73]. Okamoto et al., Nagano et al. and Zhang et al. indicated that fusible interlinings significantly increased shear stiffness [74,77,81]. The shear stiffness of face fabrics made of different weave density and adhesive interlinings made of different adhesive condition for mass, number and diameter were measured [82]. The increasing rate of shear stiffness for face fabric by bonding adhesive interlining was obtained from the difference between shear stiffness of laminated fabric and pressed adhesive interlining [83]. Figure 13 shows shearing property of (a) woollen fabric and (b) fused with interlining. It reflects that woollen fabrics become stiffer after fusing interlinings. The rapid growth of 2HG and 2HG5 values indicate the woollen fabric with interlining has a low resilience property which is due to the greatly increased inter-yarn fabrication in fabrics and inter-yarn pressure after fusing interlining [7].

#### 6.2.4. Surface

**Figure 14:**
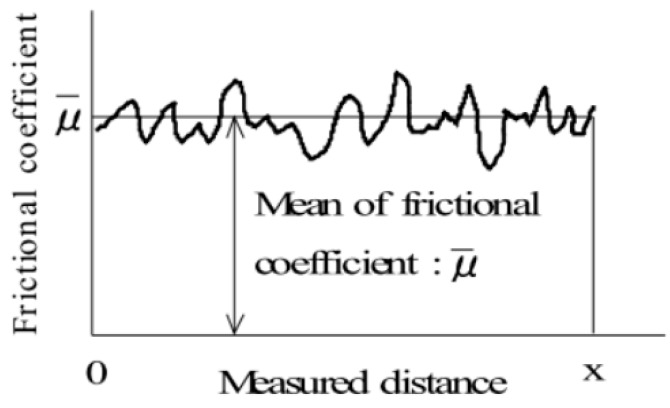
Principle of surface property measurement.

**Figure 15:**
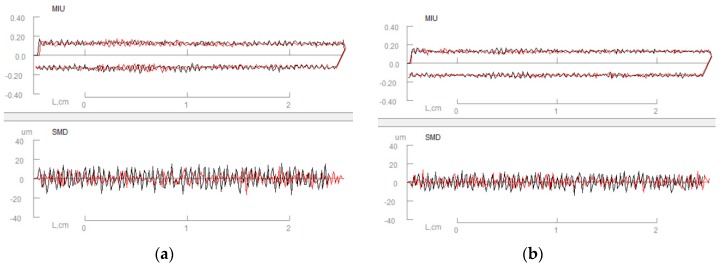
Surface property of (**a**) woollen fabric; (**b**) woollen fabric with interlining.

Surface properties include the coefficient of friction and geometrical roughness of fabrics [84]. Figure 14 shows the principle of surface property measurement [73]. It was found the free layers containing air gaps caused by raised surface fibers exhibit up to 50% higher thermal resistance than the layers joined by thermal fusion with dots of polymers [85]. Friction coefficient values increased in scatter coated nonwoven interlining because it has smoother surface so the surface resistance is increased while the sled is moving along the fabric [86]. Surface properties have not changed a lot as after shell fabric fusing interlinings it may due to the fusible interlinings are in the back of shell fabrics [7]. Figure 15 shows surface property of (a) woollen fabric and (b) fused with interlining. It can be seen that there is no significant difference between woollen fabric with and without interlining. A higher value corresponds to greater friction or more roughness features of the fabric surface. There is a slightly decrease of MIU and SMD value which shows that interlining improves softness and fullness of the woollen fabric [7]. 

#### 6.2.5. Compression

**Figure 16:**
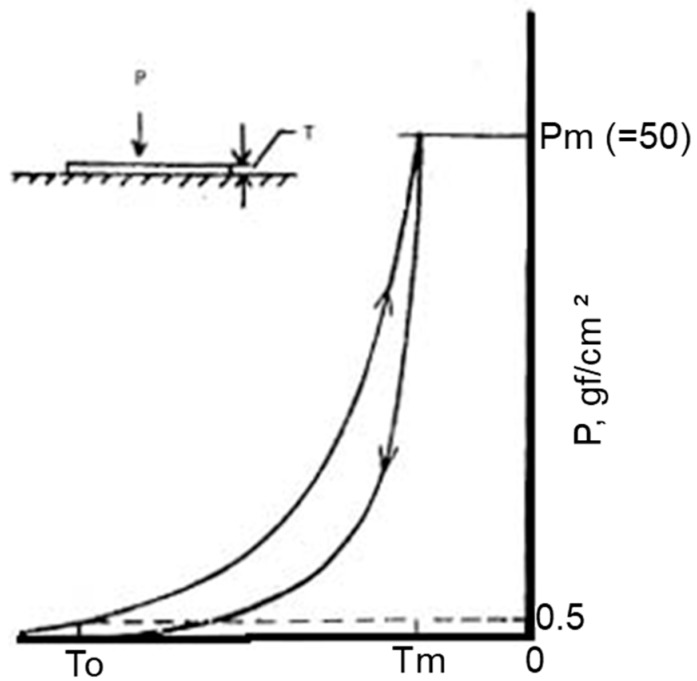
Compressional property

**Figure 17:**
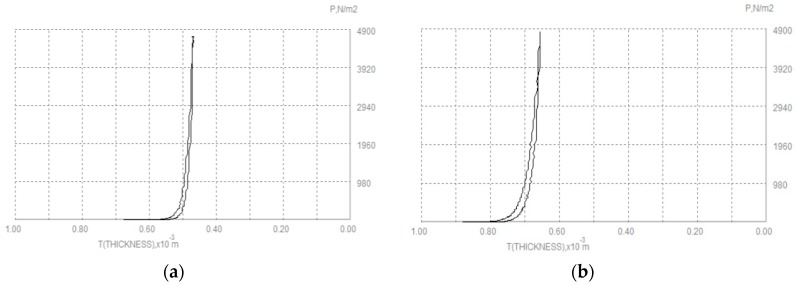
Compression property of (**a**) woollen fabric; (**b**) woollen fabric with interlining.

Compression properties include compression linearity, energy and resilience. Figure 16 shows the compressional property [73]. This reflects a fluffy and recovery of high compressibility [80]. Zhang et al. presented the fusible interlinings improve the shell fabric fluffy feeling as increasing the thickness after fusing the interlinings [7,81]. Figure 17 reflects compression property of (a) woollen fabric and (b) fused with interlining. Higher value of compression property, which shows that interlining treatment, improves the softness of woollen fabric [7]. 

## 7. Function

Interlining is used to give garments a suitable appearance and stability. The interlining is one of the most important subsidiary materials, because of its role in the final appearance and function of the garment. The fusion of interlining and face fabrics affects the style, sewing, handle comfort and fusing durability of garments. Interlinings are the key factors in the apparel accessories and play important roles in the garment’s quality [87]. Interlinings serve two major functions: One is to produce and retain the desired aesthetic appearance and the other is to improve garment quality performance [5]. Fusible interlinings in apparel manufacturing are used to support good formability, silhouette and shape retention, in another word, they are the ease of garment in order to stability of shell fabric [3]. Interlinings enhance the hand of the shell fabric and create the desired aesthetic characteristics for a garment component may be the preferred choice [4,5,88]. Interlinings are used for various purposes such as (i) Interlining is used in parts of clothing to give them thicker and firmer look, and extra strength that gives the garment more formal and appealing looks. (ii) Interlinings can improve heat insulating ability, wrinkle-proof ability and intensity of the garments, etc. [78]. (iii) Achievement of appropriate flexibility, improvement of the look, fall and applicable properties of a produced garment [89]. (iv) Interlinings are soft, flexible, and act as insulators [27], so they can be used in winter coats and pants, thus providing a thicker layer for the clothes. (v) Interlinings help to increase the efficiency of garment sewing by being easier and faster [90]. Interlining is a layer of knitted, woven or non-woven fabric placed between the garment fabrics and facing to reinforce, to give form and to prevent stretching, while the interlining type and its cut direction affect the physical and mechanical properties of the garment [27].

Some researchers investigated the degree of protection against electromagnetic (EM) radiation provided by polyamide copper-coated interlining fabric before and after dry cleaning treatment. The results obtained have shown that the reverse side of the interlining fabric had better protective properties against electromagnetic microwave radiation than the face side [21,60,61]. Phebe et al. studied the efficiency of interlining material on the comfort and dynamic deformation characteristics of body armor. The combined effect of both the fabric component and physical properties has led to an enhanced and out standing performance. Suitable plies of interlining materials were selected based on the fabric comfort properties and the impact resistance specified for ballistic application [64,90]. A considerable amount of research works has been conducted in this field. Jeong et al. studied the handle and endurance of woolen fabrics with fused interlinings and found that the bending rigidity and shear rigidity of the interlinings were most important for the handle and endurance of the clothes [61,88].

## 8. Fusing Technology

### 8.1. Fusing System

It is a fundamental requirement to fuse an interlining to an outer fabric infused during garment construction. During the early days of fusing there was very little choice in fusing equipment. Hand irons, both dry and steam were used. Steam pressing-off plant, with hand locking devices, were the main means of fusing [4]. These days there are a variety of wide and complex choices available to manufactures. There are two types of fusing systems, which are discontinuous and continuous. Details are shown in Table 3.

With fabric technology developing on a continuous basis, the requirements of fusing press technology have had to keep up. This system affects fusing by holding the assembly to be fused with a conveyor belt whilst passing through a heat source and either simultaneously or subsequently applying pressure. The advantages and disadvantages of fusing press systems are summarized in Table 4 [4,65,91,92,93,94].

### 8.2. Requirements for Fusing

The most important requirement for a fusing system is that it can provide the time, temperature and pressure necessary for fusing the fusible interlining reliably to the outer fabric.

#### 8.2.1. Processing Temperature

There is a limited range of temperatures that are effective for each fusible resin. With too low a temperature, there is an inadequate development of the adhesive properties. Too high a temperature causes the resin to become too fluid, which could produce a possible strike-back or strike-through of the resin [95,96]. In general, resin-melt temperatures can range from 130 to 160 °C, and their optimum performance will normally occur within ±7 °C of the specified temperature. This situation requires a heating source and system that they can ensure the maintenance of the selected resin-melt temperature of fusing cycles of varying times.

There are two fusing temperature should pay attention. One is upper-limit fusing temperature which the temperature that would damage the top-cloth or cause changes in color. Theoretically, this temperature would vary according to the composition of the top-cloth. However, in practice, it is rare for this temperature to exceed 174 °C (345 °F). The other is lower-limit fusing temperature which is limited because of the necessity to ensure the adequacy of the bonding required in order to withstand the handling to which fused components are subjected during production. For most clothes, this lower limit is about 110 °C (230 °F). Although leather and suede garments would require much lower temperatures because of the natural fibers which have weak thermal resistance property.

#### 8.2.2. Processing Time

The processing time starts from assemblies are actually being heated, this time temperature relates to: (i) The time when the two plates of the press actually meet with the correct specific pressure, or (ii) The time for which the assemblies are actually in the heating zone of a continuous machine is employed.

The actual fusing time can be controlled by the use of electro-mechanical timing devices or programmed card units. Generally, a flatbed press has a mechanical timer that runs to the zero point and then signals an impulse, which activates the mechanism for opening the press [97]. With continuous fusing, it is possible to relate conveyor speeds to the actual time in the heating zone. The accuracy of the timing controls can be verified by comparison with a stopwatch or by measuring the conveyor-belt speed with a tachometer [98].

#### 8.2.3. Processing Pressure

With the resins in current use, it is necessary to apply pressure to fuse in order to ensure: (i) There is an intimate contact effected between the top-cloth and fusible interlinings. (ii) The heat transfer is adequate. (iii) There is a controlled and even penetration of the resin into the fibers of the top-cloth. 

Generally speaking, the pressure of fusible interlining is highly affected by many other factors such as the device, temperature and time during the fusing system. For example, typical range of “buck” or platen pressures applied to the assembly for a flatbed fusing press is 0.2–3.0 kg/cm^2^.

#### 8.2.4. Processing Cooling

The effect of enforced cooling on fused assemblies is to achieve a consolidated bond, which enables the assembly to be handled immediately after cooling is completed. Enforced cooling can be carried out by water-cooled plates and forced-air circulation vacuum [99].

#### 8.2.5. Drying/Curing

Where binders have been used to bond the shell fabric it is necessary that the shell fabric is heated to cause the binder to cure, thereby ensuring resistance to aftercare treatment. Failure to bond the shell fabric correctly and curing the binder will lead to problems involving the shell fabric breakdown in aftercare treatments. The method of drying and curing is dependent upon the end product and its envisaged application; however, techniques such as can drying and/or hot air drying ovens are most commonly used [38,54].

## 9. Application

### 9.1. Garment Construction

Nowadays, fusible interlinings have been widely used in the garment manufacture because of the advantageous properties they have. Normally, the advantages of using fusible interlining are enabling faster assembly time, increasing strengthen, stabilize, and improving the “drape” characteristics of the garment, making it is lighter in weight and gives a soft handle as well.

There are various garments and varied instances for using different fusible interlinings. Ball et al. investigated men’s tailored jackets properties objectively and consumer perceptions subjectively. The usage of the interlining laminates, which provided a better prediction of tailor ability, especially those aspects associated with appearance and shape retention [9,43,100]. Interlinings play an important role in building shape into the detail areas of clothes, such as the fronts of coats, collars, lapels, cuffs, and pocket flaps. Also, they stabilize and reinforce areas subject to extra wearing stress, such as necklines, facings, patch pockets, waistbands, plackets, and button holes [14]. The summary of application in interlinings is shown in Table 5.

### 9.2. Drawbacks

Based on the function and application, interlining is widely used in the fashion industry. However, there are some drawbacks which can occur that are shown below.

- Strike back—Strike back is the penetration of resin to stick to the fusing press, conveyor, or shuttle tray. It can affect both the cost and the quality of fusing. It may be the result of too much resin for the type of interlining fabrication or too much pressure during the fusing process [101].
- Strike through—Strike though is the penetration of resin through to the face of the shell fabric. It may be caused by too much pressure, too high a fusing temperature, or too long a fusing time. This is a greater problem with sheer, lightweight, nonabsorbent fabrics than with heavier, bulkier, more absorbent ones. Strike through is the cause of many other problems such as color change, differential shrinkage, bubbling, poor strength, and broadness. This is a common problem with microfiber fabrics because of the construction and weight [102,103].
- Shrinkage—Shrinkage may cause performance problems if one garment part shrinks because of application of fusible interlining and adjoining pieces do not shrink. It is possible that high fusing temperature causes the fabric to shrink. This may make accurate seaming impossible, create puckered seams, or cause puckered surfaces of shell fabric. With proper testing, the amount of potential shrinkage of the shell fabric and interlining can be determined and adjustment made in patterns. During the maintenance of a garment, first of all to relaxation shrinkage and hygral extension of shell fabric and interlining in the area of fused garment parts. This means that the surface folding of stabilized garment parts can occur if the difference between the dimensional stability of shell fabric and interlining is too big [89]. Moisture content in skins varies between 14% and 18%. During the fusing operation, a loss of moisture is incurred. Each 1% change in the moisture content, represents a 1% change in the overall area of the skin. Because of this, skins cannot be subjected to the fusing temperatures employed when fusing conventional fabrics. Ideally, temperatures varying between 80 °C and 95 °C are required.
- Delamination—Delamination is the loss of bond between the interlining and shell fabric. Resin, because it migrates toward heat, becomes embedded in the interlining substrate instead of the shell fabric, which prevents an effective bond between the two materials. The shell fabric may appear to be bubbled. Delamination may be the result of under fusing, over fusing, not enough cooling time, or incompatibility of resin and the shell fabric [31].
- Color change—Color change may be a temporary or permanent discoloration caused by the high temperatures and resins used in the fusing process. Fusible interlinings with dying process may change colors when high temperatures are applied.
- Bubbling—Bubbling occurs when the face fabric or interlining becomes puckered from delamination, poor bonding, differential shrinkage, uneven temperatures or pressure, and inconsistent use of resin [89].
- Boardness—Boardness is another problem related to the inappropriate selection of adhesives used on fusible interlining. Resins liquefy and run together to form a resin coating instead of being retained in a sintered or dotted manner, a stiff hand is produced. This can be the result of over fusing, too much adhesive, and the application of excessive heat and/or pressure. This is a problem when the interlining distorts the shape of microfiber fabrics [64,104,105].

### 9.3. Interlining Selection

**Figure 18:**
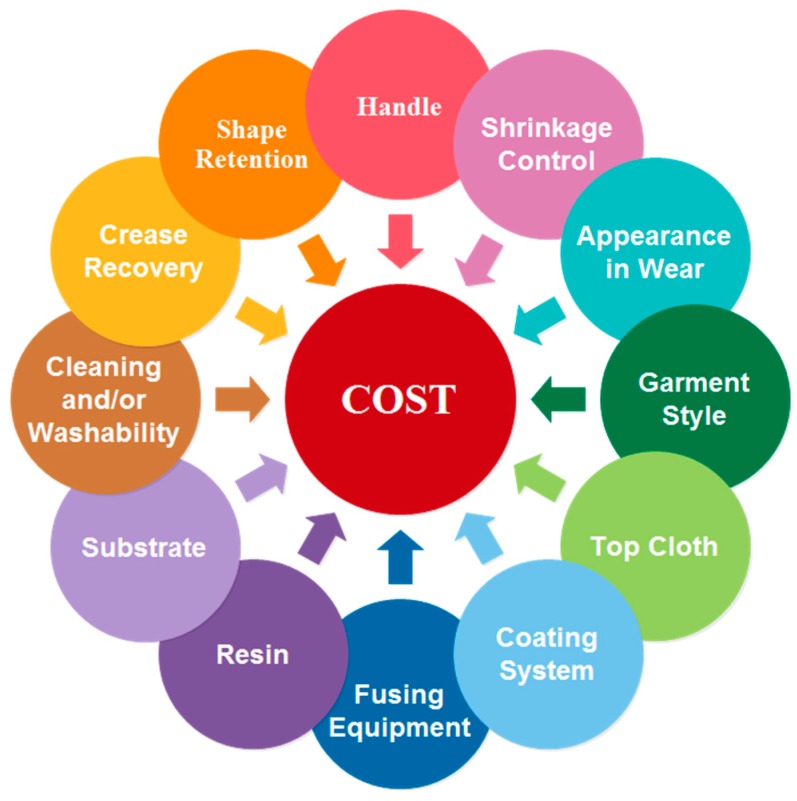
Factors influencing the choice of fusible interlinings.

The interlining fusing technique plays a very important role on the production of high-class garments, for instance, in the production of men’s suits, where it takes up more than 60 percent of the suit. This increases the importance of the matching and combination suitability of the interlining and garment face fabrics [106]. Selection of interlining with the suitable properties for the fabric and style requires knowledge of the available products, and an understanding of compatibility condition factors to be used [64]. There are some factors influencing the choice of fusible interlinings, which has shown in Figure 18. The demand from modern day production systems and techniques for consistently high quality can best be met by including the interlining selection into the early design process. At this stage the following important conditions should be established: Correct fusing conditions, desired handle, correct support, compatible, wash/dry clean resistance [88].

The correct choice of interlining is also dependent on the method of manufacture employed. It is imperative that the manufacturer heeds the suppliers’ recommendations as to the compatibility of the outer fabric with the interlining. There are numerous reports on the characteristics and performance of various fusible interlinings, and suppliers tend to recommend one kind of interlining or another for a particular application, however selecting a fusible interlining is still largely based on trial and error as well as past experience [40,74,107].

In addition, in order to achieve superior garment handle and drape in tailored garments, Fan et al. defined the desirable range of the mechanical properties of fused textile systems by integrating experimental and sensory investigations and developed a new method for proper selection of the fusible interlining for different outer fabrics [4,5,40]. Uruma and Tominomori built a system to systematically select an interlining and match it to a woolen fabric for optimal garment design. They found that fabric tensile energy, bending rigidity, bending hysteresis, shear stiffness, and shear hysteresis was significantly important for tailoring [108]. Fan et al. suggested a set of equations to predict low-stress mechanical properties of fused composites from those of composed fabric and fusible interlining fabrics [40].

The optimal arrange of adhesive interlining was considered in some studies, Jeong et al. studied a neural network and subjoined local approximation technique for selecting optimal interlinings for woolen fabrics [88]. Jeong et al. investigated the parameters of the fabrics and interlinings were selected to predict the bond quality by the neural network. The selected parameters have significant correlations in the research and the principal component analysis was used to reduce dimension of the original data [88]. The ideal composite condition range for interlining and face fabrics. Artificial neural network training was used for the structural prediction of a fused composites quality model [109,110]. Kim et al. study increasing ratio is affected by the adhesive fixing of adjacent floating yarns in addition to the fixing of interlining points. The experimentally obtained increasing ratio for a face fabric shows a linear relation with adhesive mass. It was possible to predict the shear stiffness of other laminated fabrics with the regression equation and adhesive mass [111]. Takatera et al. also predict the effects of adhesive interlining on the creep behavior of a woven fabric in the bias direction. It will be possible to predict the effects of adhesive interlining in terms of maintaining clothing shape against deformation on the bias direction. The results are also useful for the selection of a suitable adhesive interlining in garment manufacture [112]. 

## 10. New Printable Interlining

**Figure 19:**
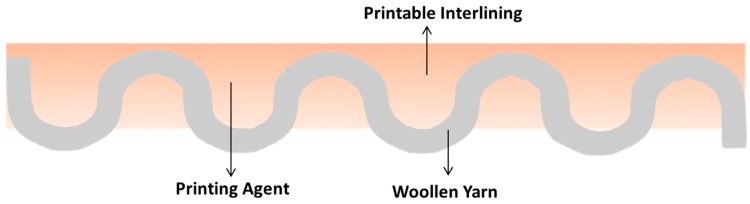
Printable interlining (Reproduced from Reference [117] with permission from Springer Nature and Copyright Clearance Center, 2018).

**Figure 20:**
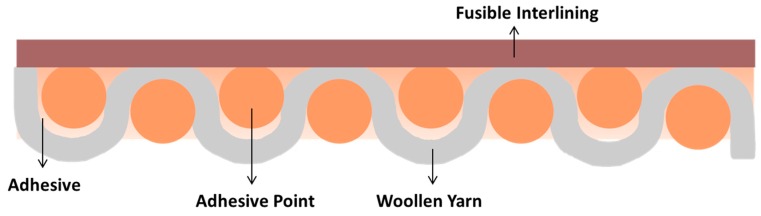
Fusible interlining (Reproduced from Reference [117] with permission from Springer Nature and Copyright Clearance Center, 2018).

A printable interlining is proposed which can be used in place of fusible interlining. Printing technique directly prints on the shell fabric and it is named printable interlining (Figure 19) which has the same function with traditional fusible interlining (Figure 20) in terms of physical and mechanical properties and hand value. The motivation of printable interlining is the drawback of traditional fusible interlinings which are costly and involve a tedious production process, and they have some quality problems such as strike through/back and bubbles. There is an obvious advantage of printable interlinings comparing with fusible interlinings in terms of manufacture operation, quality problems, material using and the thickness/weight of interlining details shown in the Table 6. 

Printing is a cost-effective and scalable technology with high throughput and is highly compatible with simple processing, which facilitates mass production at an extremely low cost [8,10,113,114]. Considering this technology, the advantage of screen-printing with a rapid apparatus, various pattern and flexible size change of specimens for experimental operations which is suitably used for experiments [115]. The variations of experimental conditions, such as agent, screen mesh, as well as stroke frequency, have given some conclusive results in changing the total hand value and mechanical properties of woollen fabrics. Fabric quality is generally perceived through fabric hand value [116]. A series of laboratory experiments carried out to compare total hand value and low-stress mechanical properties (tensile, bending, shearing surface and compression) of traditional fusible interlinings and printable interlinings by KESF. The result of this study is to prove printable interlinings can replace traditional fusible interlinings by achieving a better performance on woollen fabrics. Printable interlinings show a significant development of total hand value and increase the fabric stiffness, shearing banding and tensile properties comparing with fusible interlinings [117]. The new printable interlining enhances quality and reduces operational process and production cost for garment manufacturers. It can be widely used in mass production with a simple control process and it is cost-effective [117]. This was not only improved garment manufacture with easy and cost-effective process but also developed the hand value and low-stress mechanical properties of woollen fabric.

## 11. Conclusions

Fusible interlinings are currently enjoying a rapid pace, with enormous research achievements currently emerging. The functions of fusible interlinings conducted to improve the shell fabric for the quality, and aesthetic performances. These advantages will endow garments with various design styles and develop apparel manufacturing. This review article focused on different kinds of fusible interlinings and compared their usage requirements. There are various fusible interlining technologies issues that were covered, and the production process has been explored as well. Updated discussions of the advantages and disadvantages of the different kinds of interlinings that have been used up to now can be utilized for a better understanding of how the structure and technique related to interlinings can be handled in application.

Afterwards, special attention has been raised focusing on the functions and applications of fusible interlinings. Novel printable interlinings were proposed for the development of interlining which in place of traditional fusible interlining in garment manufacture. Printable interlinings are newly invented which have similar or better properties and have a high-value performance compared with fusible interlinings. The printable interlining has a cost-effective and simple process for garment manufacture. Finally, the interlining concept of multi-fabrics for garment design and manufacture has good and variable manifestations.

## Figures and Tables

**Table 1:** Summary of interlinings’ evolution.

Years	Milestones	Author/Reference
1937	Sewing interlining to prevent garment losing its shape and neat appearance obtained when freshly laundered.	Lind, Jay H. Apparel and parts thereof. U.S. Patent No. 2,102,014. 14 December 1937 [11].
1955	Non-woven textile-like sheet material which is porous, air-permeable, crease-resisting, shrink-proof fibrous sheet material. It was capable of being sewed, stitched or padded.	Ludwig, Nottebohm Carl. Fibrous, non-woven textile-like sheet material. U.S. Patent No. 2,719,802. 4 October 1955 [12].
1956	A so-called fused fabric assembly that is semi-stiff fabric composed of two or more separately manufactured fabric plies. The fabric is superimposed upon one another by means of thermoplastic elements contained.	Lajos, Bihaly. Fused fabric assemblies. U.S. Patent No. 2,757,435. 7 August 1956 [13].
1962	Improving the fabric durability, hand, softness, insulating capacity, bulk and softness.	Carlyle, Harmon, William S. Barnard, and Cecil E. Johnson. Method of manufacturing an insulating interlining fabric. U.S. Patent No. 3,043,733. 10 July 1962 [14].
1969	A fusible interlining material is produced by adhering to a base fabric. The adhesive thermoplastic net is heated and soften able on localized areas.	Barb, Wolfgang Gerson. Method of forming a fusible interlining material. U.S. Patent No. 3,464,876. 2 September 1969 [15].
1972	A fusible resin, distributed as a myriad of closely spaced small dots, is employed to unite the two fabric layers	Miller, Samuel E. Interlining. U.S. Patent No. 3,703,730. 28 November 1972 [16].
1975	A crosslinking agent for the resin which contacts with the resin particles	Lauchenauer, Alfred E. Heat-sealable interlining for textile fabrics. U.S. Patent No. 3,922,418. 25 November 1975 [17].
1978	An interlining structure of apparel using waterproofed outer fabrics	Sato, Koichi. Interlining structure for apparel and method for its production. U.S. Patent No. 4,076,881. 28 February 1978 [18].
1983	A fusible interlining having enhanced bond strength with resistance to dry cleaning and comprising a support web and a heat actively adhesive	Kamat, Dattatraya V. Fusible interlining of improved bond strength and dry cleaning resistance. U.S. Patent No. 4,415,622. 15 November 1983 [19].
1984	Fused and needled nonwoven interlining fabric	Knoke, Jurgen, et al. Fused and needled nonwoven interlining fabric. U.S. Patent No. 4,490,425. 25 December 1984 [20].
1985	Method for manufacturing an adhesive interlining with component of two layer non-woven fabric	Ohta, Nobuo. Method for manufacturing an adhesive interlining and fabric produced thereby. U.S. Patent No. 4,511,615. 16 April 1985 [21].
1994	Non-woven interlining substrate with a single or double bar ripple weft stitch knitted there through	Longo, Pietro. “Non-woven interlining.” U.S. Patent No. 5,294,479. 15 March 1994 [22].
2001	Crosslinking base layer for bondable interlinings in accordance with the double dot tech technique	Simon, Ulrich, Thorsten Gurke, and Hans-Willi Losensky. Crosslinking base layer for bondable interlinings in accordance with the double dot tech technique. U.S. Patent No. 6,300,413. 9 October 2001 [23].
2004	A microencapsulated adhesive component in a cross-linkable hot-melt adhesive component for coating and/or laminating surface formations was a development	Simon, Ulrich, and Guenther Koehler. Crosslinking base layer for fixing interlinings according to double dot and paste process. U.S. Patent No. 6,784,227. 31 August 2004 [24].
2006	A fusible interlining includes a textile interlining web coated with a plurality of double-layered adhesive dots on a first side of the textile interlining web	Morris, Paul; Horsfield, Michael. An interlining material, process of manufacturing and use thereof. U.S. Patent Application No. 11/488,413, 2006 [25].

**Table 2:** Low-stress mechanical properties in KES-F system [73].

Properties	Symbol	Definition	Characteristics	Unit
Tensile energy /tensile work	WT	Energy used for extending fabric to 500 gf/cm width.	WT refers to the ability of a fabric to withstand external stress during extension. Fabric with good tensile strength and toughness will have a large value of WT.	gf.cm/ cm^2^
Tensile resilience	RT	Percentage energy recovery from tensile deformation.	The reduced fabric RT value implies that the fabric becomes difficult to restore to its original shape after releasing the applied tensile stress.	%
Tensile extensibility	EMT	Percentage extension at the maximum applied a load of 500 gf/cm specimen width.	EMT has a good correlation with fabric handle. The greater the value of EMT, the larger the elongation of the fabric under a known applied stress will be.	%
Tensile linearity	LT	Linearity of load extension curve	LT is a measure of the deviation of the load extension curve straight line.	-
Shear stiffness/shear rigidity	G	The average slope of the linear regions of the shear hysteresis curve to ±2.5° shear angle.	G refers to the ability of a fabric to resist shear stress which is the ease with which the fibers slide against each other. Lower values indicate less resistance to shearing corresponding to a softer material having better drape.	gf/cm ·degree
Shear stress at 0.5^o^	2HG	The average width of the shear hysteresis loop at the ±0.5° shear angle.	2HG is the ability of a fabric to recover after applying the shear stress value of 0.5° shear angle. The greater the value of shear stress, the worse the recovery ability of the fabric and stiffer the fabric will be.	gf/cm
Shear stress at 5^o^	2HG5	The average width of the shear hysteresis loop at the ±5° shear angle.	2HG5 is the ability of a fabric to recover after applying shear stress value of 5° shear angle. The greater the value of shear stress, the worse the recovery ability and stiffness of the fabric will be.	gf/cm
Bending rigidity	B	Average slope of the linear regions of the Bending hysteresis curve to 1.5 cm^−1^.	B is the ability of a fabric to resist the bending moment, which is related to the quality of stiffness when a fabric is handled. Higher B value indicates greater resistance to be bent.	gf.cm^2^/cm
Bending moment	2HB	The average width of the bending hysteresis loop at 0.5 cm^−1^ curvatures.	2HB refers to the recovery ability of a fabric after being bent. It is measured as a specimen is bent through a range of curvatures from 2.5 cm^−1^ to −2.5 cm^−1^. The smaller the value of 2HB, the better the bending recovery of the fabric will be.	gf.cm/ cm
Compressional linearity	LC	The linearity of a compression-thickness curve.	LC determines the compressibility along with the change in fabric thickness after treatment. The high value of LC indicates a fluffy fabric with high compressibility.	-
Compressional energy	WC	Energy used for compressing fabric under 50 gf/cm^2^.	The WC value represents a fluffy feeling of the fabric. The higher the value of WC, the higher the compressibility of the fabric will be.	gf.cm/ cm^2^
Compressional resilience	RC	Percentage energy recovery from lateral compression deformation.	RC indicates the recoverability of the fabric after the compression force is removed. A higher value indicates better recovery ability from compression.	%
Fabric thickness at 0.5 gf/cm^2^ pressure	T_O_	Fabric thickness at 0.5 gf/cm^2^ pressure.	T_O_ measures the surface thickness at a pressure of 0.5 gf/cm^2^.	mm
Fabric thickness at 50 gf/cm^2^ pressure	T_M_	Fabric thickness at 50gf/cm^2^ pressure.	T_M_ measures the intrinsic thickness at a pressure of 50 gf/cm^2^.	mm
Coefficient of friction	MIU	The coefficient of friction between the fabric surface and a standard contactor.	MIU represents the fabric smoothness, roughness, and crispness. The value demonstrates the ratio of the force required to slide the surfaces to the force perpendicular to the surfaces. The higher the value of MIU, the greater the friction of the fabric will be.	-
Geometrical roughness	SMD	Variation in surface geometry of the fabric.	SMD refers to the fabric surface evenness. The lower the SMD value, the more even the fabric surface will be.	micron
Mean deviation of MIU	MMD	Mean deviation of coefficient of friction	MMD is the frictional measurement.	-

**Table 3:** Types of fusing systems available.

Discontinuous	Continuous
Hand iron	Horizontal
Steam press	Rotary drum
Flat bed press	
High frequency fusing	

**Table 4:** Summary of advantages and disadvantages of fusing press systems.

	Introduction	Advantages	Disadvantages
**Hand iron**	This system effects fusing by placing a hand iron on top of the assembly to be fused (interlining uppermost). Certain interlinings, especially those that can be fused at comparatively low temperatures, low pressure and short times, may be suitable for fusing by hand iron.	Maneuverability Low cost	Poor pressure maintenance Poor heat control should be limited to edge tape and work aids
**Steam press**	This system effects fusing by placing the assembly to be fused between the platen of a steam utility press normally used for intermediate or finishing-off pressing of made-up garments.	Good heat penetration Dual purpose-can be used for fusing or off pressing Vacuum can be used for fusing or cooling	Poor pressure control Attention to lagging to maintain steam pressure Low throughput Poor operator environment
**Flat bed press**	These system effect fusing by placing the assembly to be fused between metal platen, one or both of which can be heated.	Space saving Low cost Positive pressure during entire fusing cycles, resulting in low heat shrinkage	Possibility of high strike through and back Heat on one surface only Sandwich fusing not recommended Table models suitable for small part fusing only Possibility of moisture entrapment Operator exposed to heat
**High frequency fusing press**	This system affects the fusing of the assembly by applying high frequency waves to the materials, which are placed between the high frequency generating top and bottom plates.	Very good temperature control Cool working conditions High throughput Low running cost Low thermal shrinkage	High initial cost Possible strike back Currently suitable for high production only Cannot be used on fabrics with metal content or allow any metal to enter press Evenness and stack height critical
**Continuous horizontal press**	These systems affect fusing by holding the assembly to be fused with a conveyor belt whilst passing through a heat source and either simultaneously or subsequently applying pressure.	Minimal strike through and back Some cooling available on an exit conveyor The press is heated top and bottom, using sophisticated temperature control Ability to sandwich fuse High output due to continuous nature of machine Cooling and stacking facilities easily applied Pre-heat and pressure available on some models	Higher heat shrinkage than with flat bed press Belts and pressure rollers susceptible to wear and damage Difficult to monitor to faulty heating element on some models
**Continuous drum type press**	These systems effect fusing by holding the assembly to be fused with a conveyor belt whilst passing through a heat source and either simultaneously or subsequently applying pressure.	One operator system Space saving on table models Low initial cost Some models available in single phase	Pressure springs prone to weaken if fitted Suitable for small part fusing only Poor cooling facilities on some models Poor pressure control on some models Possible to set fused assembly into curved state

**Table 5:** Summary of application for interlining.

Suit Coats and Blazers	Coats	Wool Sport Outerwear	Dresses	Shirts	Trousers	Blouses
Entre coat front Facing-lapel piece Top and bottom collar Cuff wigan Flaps, tabs, welts, vents, bottom, bands, yokes Chest piece Felt piece Shoulder piece Pocket path, stay piece Bridle Tapes	Entire fused front Entire facing Cuffs Packets, flaps, tabs, belts, vents, yokes, bottom bands Packets Epaulets Collars-top and bottom Sleeve heads Special support fabric for fur collars Chest piece or felt piece Warmth insulation Trapunto fillers	Entire coat front Entire Facing Lapel piece Cuffs Chest piece and felt piece Collars-top and bottom Edge tapes Epaulets, belts Flaps, tabs, vents, yokes, bottom bands Special fillers	Collars Facings-closed or open Cuffs Pockets, flaps, tabs Welts, vents, yokes Plackets Epaulets and belts Pleating and belts Neck facing Waist bands Edge tapes	Collars-top or top and bottom Facings Plackets Cuffs ¼ Panels Neck Bands	Waistbands Fly linings Extension tabs Pocket flaps Pocket patches Belt loop filler	Collars-top or bottom or both Facings Packets Cuffs Tapes Pleat Stabilization

**Table 6:** Comparison of printable interlining and fusible interlining.

Comparison	Printable Interlining	Fusible Interlining
Method	Print on the fabric directly	Interlining attached by fusing
Operation	Easy	Difficult
Quality Problems	Less	More
Material Using	No Need	Need
Thickness/Weight	Easy to vary	Not easy to vary
